# Unveiling the role of ABI3 and hub senescence-related genes in macrophage senescence for atherosclerotic plaque progression

**DOI:** 10.1007/s00011-023-01817-w

**Published:** 2023-12-07

**Authors:** Yajuan Fu, Juan Zhang, Qiujun Liu, Lan Yang, Qianqian Wu, Xiaomin Yang, Lexin Wang, Ning Ding, Jiantuan Xiong, Yujing Gao, Shengchao Ma, Yideng Jiang

**Affiliations:** 1 National Health Commission Key Laboratory of Metabolic Cardiovascular Diseases Research, Ningxia Medical University, Yinchuan, China; 2 Ningxia Key Laboratory of Vascular Injury and Repair Research, Ningxia Medical University, Yinchuan, China; 3 Department of Pathophysiology, School of Basic Medical Sciences, Ningxia Medical University, Yinchuan, China; 4 School of Laboratory Medicine, Ningxia Medical University, Yinchuan, China

**Keywords:** Atherosclerosis, Macrophage senescence, Machine-Learning, Biomarkers, Sc-RNA seq

## Abstract

**Background:**

Atherosclerosis, characterized by abnormal arterial lipid deposition, is an age-dependent inflammatory disease and contributes to elevated morbidity and mortality. Senescent foamy macrophages are considered to be deleterious at all stages of atherosclerosis, while the underlying mechanisms remain largely unknown. In this study, we aimed to explore the senescence-related genes in macrophages diagnosis for atherosclerotic plaque progression.

**Methods:**

The atherosclerosis-related datasets were retrieved from the Gene Expression Omnibus (GEO) database, and cellular senescence-associated genes were acquired from the CellAge database. R package Limma was used to screen out the differentially expressed senescence-related genes (DE-SRGs), and then three machine learning algorithms were applied to determine the hub DE-SRGs. Next, we established a nomogram model to further confirm the clinical significance of hub DE-SRGs. Finally, we validated the expression of hub SRG ABI3 by Sc-RNA seq analysis and explored the underlying mechanism of ABI3 in THP-1-derived macrophages and mouse atherosclerotic lesions.

**Results:**

A total of 15 DE-SRGs were identified in macrophage-rich plaques, with five hub DE-SRGs (ABI3, CAV1, NINJ1, Nox4 and YAP1) were further screened using three machine learning algorithms. Subsequently, a nomogram predictive model confirmed the high validity of the five hub DE-SRGs for evaluating atherosclerotic plaque progression. Further, the ABI3 expression was upregulated in macrophages of advanced plaques and senescent THP-1-derived macrophages, which was consistent with the bioinformatics analysis. ABI3 knockdown abolished macrophage senescence, and the NF-κB signaling pathway contributed to ABI3-mediated macrophage senescence.

**Conclusion:**

We identified five cellular senescence-associated genes for atherogenesis progression and unveiled that ABI3 might promote macrophage senescence via activation of the NF-κB pathway in atherogenesis progression, which proposes new preventive and therapeutic strategies of senolytic agents for atherosclerosis.

## Introduction

Atherosclerosis is the main pathological basis for subsequent cardiovascular complications, including ischemic heart failure and stroke, making it the leading cause of mortality worldwide [1]. Atherosclerosis is initiated by endothelial cell injury, followed by a cascade of events such as lipid layer or fatty streak formation, leukocyte and smooth muscle cell migration, foam cell formation and extracellular matrix degradation within the large arteries. Major stages of plaques including fatty streak, plaque progression and plaque disruption, have been identified [2]. Advanced plaques are more fragile and susceptible to rupture, which can lead to the formation of abrupt thrombus and trigger cerebrovascular disease. Invasive intravascular imaging are widely used to evaluate vessel stenosis and wall thickness, but it is still limited in identifying stable cardiovascular atherosclerotic disease [3, 4]. Therefore, developing tools for the diagnosis of early-stage and advanced atherosclerosis at a biochemical, cellular or molecular level is desiderated.

Atherosclerosis is closely related to ageing, and senescent cells are more abundant in chronic diseases. Cellular senescence is characterized by irreversible arrest of the cell cycle, distinctive alteration of phenotype (flattened and enlarged morphology) and metabolic reprogramming (especially senescence-associated secretory phenotype (SASP)). Meanwhile, the expression of senescence-related proteins such as p53, p21 and p16 are upregulated and the activity of senescence-associated β-galactosidase (SAβG) is increased [5]. Evidence has accumulated that senescent vascular cells , including endothelial cells, vascular smooth muscle cells (VSMCs) and macrophages, are involved in the progression of atherosclerotic lesions [6, 7]. Moreover, advanced plaques are inundated with senescent cells, which can not only drive the maturation of atherosclerotic lesions via inflammation and monocyte chemotaxis but also accelerate the degradation of the extracellular matrix [8]. Accumulating evidence suggests that macrophages could act as a driver in all stages of atherogenesis (from “fatty streak” to complex plaques), and senescent foamy macrophages contribute to early atherogenesis and complex advanced lesions. Furthermore, the removal of senescent macrophages stabilized atherosclerotic plaques by reducing inflammatory cytokines, monocyte recruitment factors and plaque destabilization-related matrix metalloproteases [8, 9]. However, the role of senescent macrophages in atherogenesis remains mysterious, as studies have found that mice deleting senescence-related genes p21 or p19 (ARF) showed aggravated atherosclerosis [10, 11].

This study aims to explore differentially expressed senescence-related genes (DE-SRGs) based on the gene expression microarray datasets from the GEO database. Three machine-learning algorithms were employed to screen hub DE-SRGs, and a nomogram model was established to determine the diagnostic capacity of the hub DE-SRGs for the progression of atherosclerotic plaques. We also developed early and advanced plaques in Apolipoprotein E-knockout (*ApoE*^*−/−*^) mice to validate the expression of ABI3, one of the hub DE-SRGs, in macrophages of the plaques. The results revealed that ABI3 was upregulated in advanced plaques and participated in homocysteine-induced macrophage senescence through the NF-κB signal pathway. This study provides new insights into preventing or delaying atherosclerosis progression and other aging-related diseases.

## Materials and methods

### Data acquisition

The microarray datasets GSE163154, GSE41571 and GSE43292 were obtained from the GEO database (https://www.ncbi.nlm.nih.gov/geo/). The GSE163154 series [12] contains 42 carotid artery lesions (including 26 intraplaque hemorrhage (IPH) and 16 non-IPH samples) and GSE41571 consists of 5 ruptured and 6 stable plaques excised from macrophage-rich regions [13] were selected as test sets. Microarray expression data of early-stage and advanced plaques from 32 patients carotid plaques in the GSE43292 dataset [14] was employed to validate the common shared gene expression levels. The genes associated with cell senescence were acquired from CellAge database (https://genomics.senescence.info/cells/) [15].

### DE-SRGs screen

The series matrix files were downloaded from the GEO database and normalized with robust multi-array average (RMA) method [16] by R version 3.6.3 software. The expression data of 279 SRGs were extracted from GSE163154 and GSE41571, and “limma” R package was employed to distinguish the DE-SRGs (*P* < 0.05& |log fold change (FC)|> 0.5). The DE-SRGs were visualized with volcano plots via “ggplot2” package and the common DE-SRGs between the two datasets were overlapped through the venn diagram.

### Identification of hub DE-SRGs via machine learning algorithms

Three machine learning algorithms, including Least Absolute Shrinkage and Selection Operator (LASSO), Support Vector Machine-Recursive Feature Elimination (SVM-RFE), and Random Forest (RF), were employed to screen hub DE-SRGs. LASSO analysis was implemented with the R package “glmnet” [17] with 10 cross-validation to screen the optimal tuning parameter (λ). The SVM-RFE algorithm was utilized to select the point with the smallest cross-validation error to determine the variable through packages “e1071” and “caret” [18]. The package “randomForset” was employed to develop a random forest model, and top 10 DE-SRGs with MeanDecreaseGini score were chosen [19]. Finally, a Venn diagram was used to visualize the hub DE-SRGs intersected from the three algorithms.

### Immune cell infiltration evaluation and gene set enrichment analysis (GSEA) analysis of hub DE-SRGs

The immune enrichment scores of 28 immune cell were calculated through the single-sample gene set enrichment analysis (ssGSEA) method and their correlation with hub DE-SRGs were visualized with R package “corrplot”. We obtained gene set h.all.v7.4.entrez.gmt from MSigDB (http://www.gsea-msigdb.org/gsea/msigdb/index.jsp) [20], and GSEA analysis was conducted based on the expression levels of ABI3 via R package “clusterProfiler”.

### Diagnostic performance of hub DE-SRGs

To assess the diagnostic efficiency of the identified hub DE-SRGs, the receiver operating characteristic (ROC) curves of each gene and all genes contained in one diagnostic model were separately plotted with the R package “pROC” [21]. Meanwhile, a nomogram was developed using the R package “rms” [22], and the calibration, decision and clinical impact curves were also established based on the hub DE-SRGs.

### Single-cell RNA-seq data analysis

The scRNA-seq data (GSE205930) of atherosclerosis were obtained from the GEO database and imported into R for analysis using the Seurat V4.1.0 package [23]. We eliminated cells with the following criteria: a gene count per cell  between 200 and 3000, and a percentage of mitochondrial genes less than 20%. Following quality control, the data was normalized and the top-ranked 2000 variably expressed genes were selected. Prior to Principal Component Analysis (PCA), the data was scaled using the ScaleData function. Then a combination of methods including “RunHarmony”, “FindNeighbors”, “FindClusters” (with a resolution parameter set from 0.2 to 1.0 with an interval of 0.1), and the “RunUMAP” functions were employed to perform unbiased and unsupervised clustering of the cell subpopulations. Subsequently, the cell clusters and expression of ABI3 were visualized using the “scCustomize” package. For evaluating gene signature scores, “UCell” package [24] was employed based on mh.all.V2023.1.Mm.symbols.gmt collected in the GSEA database.

### Animal models and aortic tissue collection

Male *ApoE*^−/−^ mice were purchased from the Animal Center of Peking University Health Science Center (Beijing, China), and the experiments were approved by the Institutional Animal Care and Use Committee of Ningxia Medical University (IACUC-NYLAC-2021-038). Mice were fed with high-methionine diet for 4 weeks or 18 weeks to induce early or advanced lesions as previously reported [25]. Different treated mice were anesthetized, and blood was collected from the orbital sinus, and aortic tissues were collected and frozen for assessing atherosclerotic lesions and gene expression.

### Evaluation of atherosclerotic lesions

Frozen aortic tissues of different treated mice were embedded in OCT, tissue sections were prepared as previously described [26], and stained with H&E (BiYuntian, Shanghai, China). The cryostat aortic tissue sections were dipped in 60% isopropanol, followed by staining with 2 mg/mL Oil Red O solution at room temperature. Images were captured with a microscope (Leica, Germany). The extent of atherosclerotic lesions in the aorta and aortic sinus was analyzed using Image J software.

### Immunofluorescence staining

The aortic roots of *ApoE*^−/−^ mice were fixed in 4% paraformaldehyde for 20 min and then were permeabilized with 0.5% Triton X-100 for 20 min. Following blocking with 3% BSA for 1 h, the specimens were incubated overnight at 4 °C with specific primary antibodies against ABI3, p53 or F4/80 (a marker of macrophages). After incubation with secondary antibodies (FITC-and ABflo 647-conjugated) for 1 h at room temperature, and then the specimens were counterstained with DAPI to visualize the nucleus. Digital images were captured using a confocal laser scanning microscope (LSM800; Zeiss, Oberkochen, Germany).

### Cell culture

THP-1 cells, a human monocytic leukemia cell line (Chinese Academy of Life Sciences, Shanghai, China), were maintained in RPMI 1640 (GIBICO, USA) medium with 10% fetal bovine serum (FBS), 100 μg/mL streptomycin and 100 U/mL penicillin in a 5% CO_2_ incubator at 37 °C. Cells were differentiated into macrophages by 100 nmol/L PMA (Sigma-Aldrich, St Louis, USA) induction for 24 h, followed by culture in fresh medium supplemented with Hcy for another 48 h.

### Small interfering RNA (siRNA) transfection

Gene knockdown was carried out using the HiPerFect Transfection kit (Qiagen, Germany). THP-1 cells were transfected with 5 nM siRNA against ABI3 following the protocol of manufacturer, and cells transfected with irrelevant non-targeting siRNA, or were treated with sham transfection were used as negative control. The cells were cultured for another 24 h after transfection, and gene knockdown efficiency was measured by qRT-PCR and western blot.

### qRT-PCR

RNA was extracted from the aorta tissue and macrophage cells using Trizol reagent (Invitrogen), and cDNA synthesis was performed with a reverse transcription kit (Takara, Dalian, China). The expression of ABI3 was detected by qRT-PCR with SYBR green reagents (MBI, Vilnius, Lithuania). Primers used were as follows: ABI3 Forward, ATCGCCCCAGAGAACCTACC; ABI3 Reverse, GCTCTTTCGAGACAGGGTGC; β-actin Forward, CATGTACGTTGCTATCCAGGC; β-actin Reverse, CTCCTTAATGTCACGCACGAT. All experiments were carried out in triplicates.

### Western blot

Tissues and cells were lysed using RIPA lysis buffer, and western blot was performed according to the methods previously established [27]. In brief, an amount of 30 μg proteins were loaded and separated by 12% SDS-PAGE and then transferred to the PVDF membrane. After blocking, membrane were incubated with anti-ABI3 and anti-GAPDH antibodies and HRP-conjugated goat anti-mouse IgG secondary antibody. Protein bands were visualized using the Gel Documentation and Analysis System ChemiDoc XRS system with the chemiluminescence kit (Biyuntian, Shanghai, China).

### Senescence-associated β-galactosidase (SA-β-Gal) staining analysis

To assess cellular senescence, SA-β-Gal staining was performed according to a modified protocol [28]. Cells were fixed with 4% paraformaldehyde and incubated at 37 °C overnight with X-gal (Solarbio, China) following the manufacturer's instructions. After mounting coverslips, images were captured using a microscope (Leica, Germany) at a magnification of 20 × to quantify SA-β-gal-positive cells.

## Statistical analysis

Statistical analysis was performed using two-tailed unpaired Student’s *t* test (two-groups comparison) and One-way ANOVA (multiple-groups comparison) with GraphPad Prism Version 8, US. All data are expressed as mean ± S.D. and statistical significance was considered when *P* < 0.05.

## Results

### Identification of differentially expressed senescence-related genes (DE-SRGs) in atherosclerotic plaque progression

**Fig. 1:**
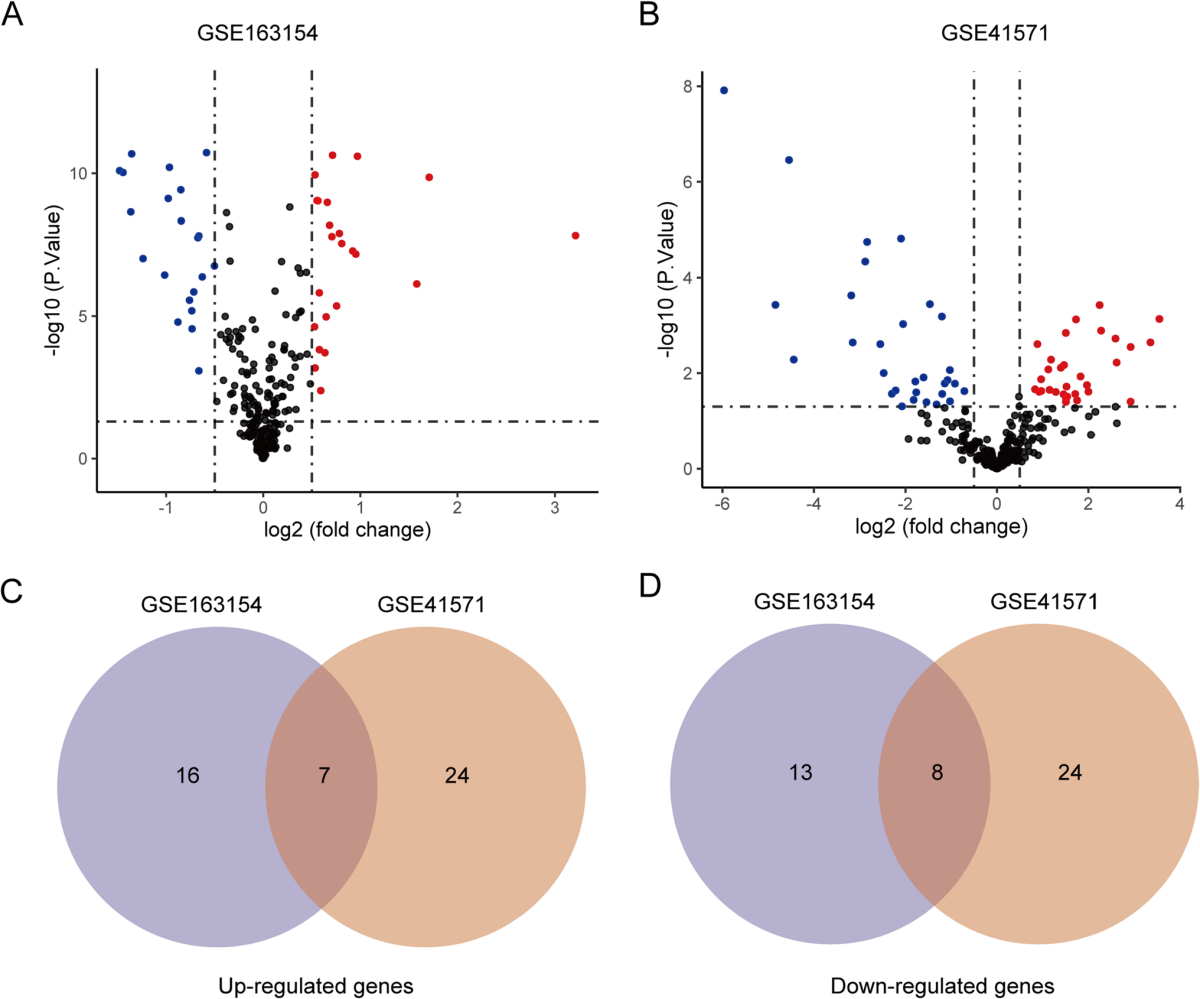
Expression features of SRGs in atherosclerotic plaque progression. Volcano plots of SRGs in **A** GSE163154 and **B** GSE41571 datasets. The Venn diagram illustrates the common **C** up-regulated and **D** down-regulated differentially expressed SRGs between the two datasets

Cellular senescence is implicated in atherosclerosis and contributes to features of plaque instability. To investigate the SRGs signature in atherosclerotic plaque progression, microarray profiles were retrieved from the GEO database, and 279 SRGs were obtained from the CellAge database. R software (R 4.1.2) was utilized to normalize and logarithmize the series matrix files, and DE-SRGs were screened using a criterion of (*P* < 0.05&|logFC|> 0.5). A total of 257 SRGs including 23 up-regulated and 21 down-regulated DE-SRGs in advanced plaque compared with stable plaque, were identified in GSE163154 (Fig. 1A). Similarly, 259 SRGs were extracted from GSE41575, among which 31 genes were up-regulated and 30 genes were down-regulated in advanced plaque according to the volcano plot analysis (Fig. 1B). Consequently, 15 common DE-SRGs were found to be overlapping between the two datasets (Fig. 1C and D), implying the involvement of these genes in atherosclerotic plaque progression.

### Determination hub DE-SRGs via machine learning algorithms

**Fig. 2:**
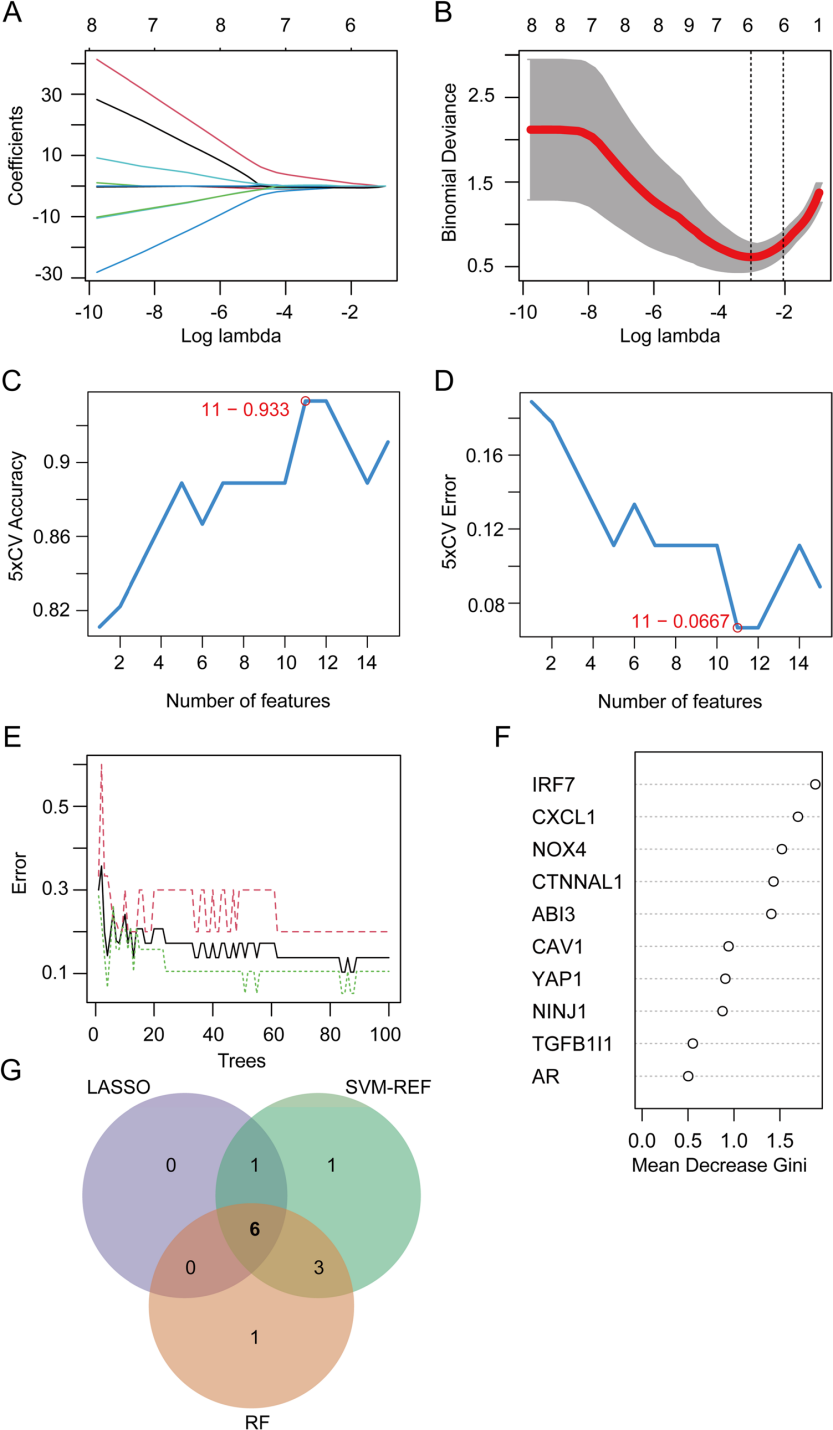
Hub DE-SRGs screening based on machine learning algorithms. **A**, **B** LASSO regression model was established based on the 15 DE-SRGs. **C**, **D** SVM-REF model for feature selection. **E**, **F** Random Forest (RF) model showed the top 10 DE-SRGs in terms of importance. **G** Venn diagram showed the six intersected hub DE-SRGs (ABI3, CAV1, IRF7, NINJ1, Nox4 and YAP1) shared by LASSO, SVM-RFE and RF algorithms

**Fig. 3:**
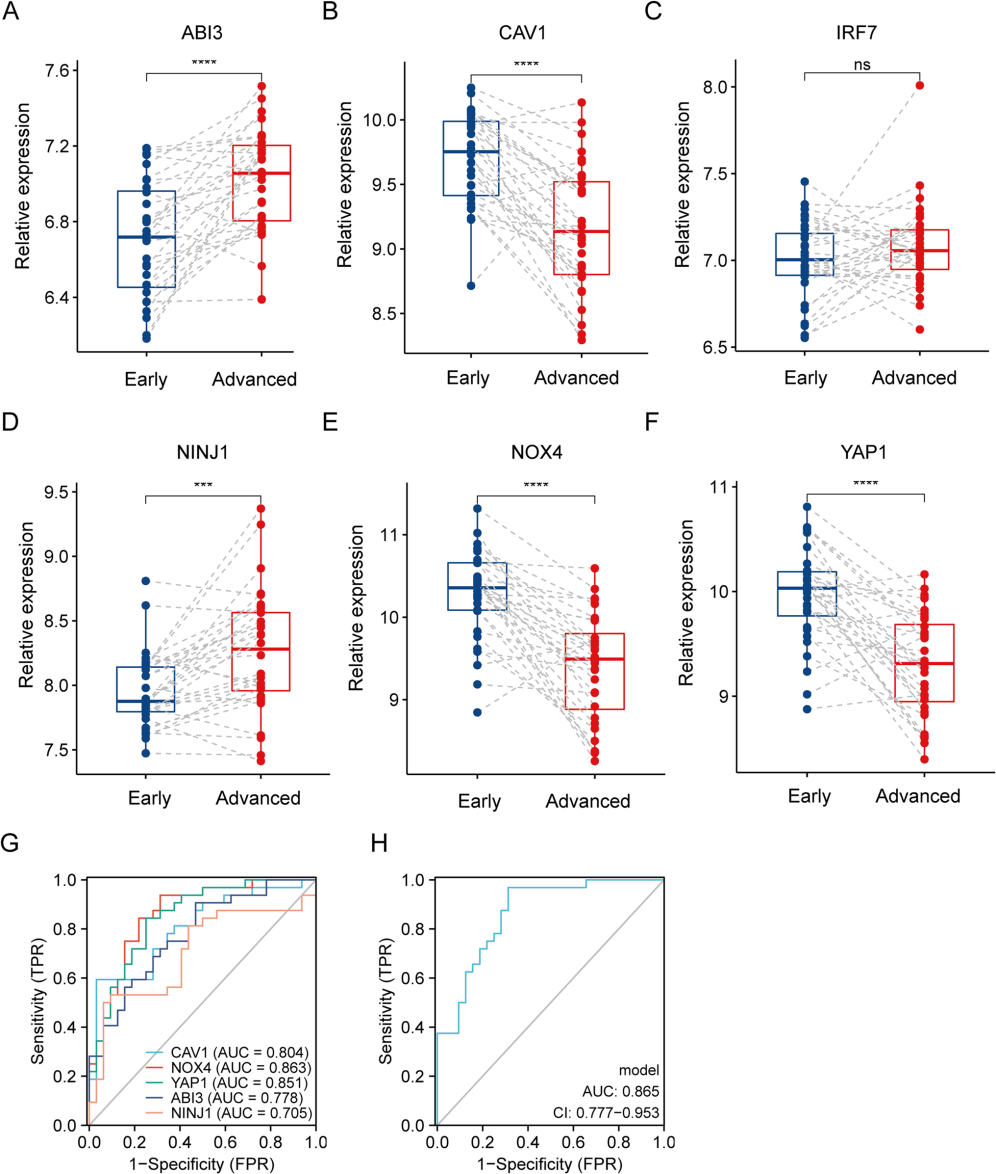
Validation of the hub DE-SRGs and their diagnostic capacity. **A**–**F** The expression levels of the five hub DE-SRGs, ABI3, CAV1, NINJ1, Nox4, and YAP1 in early and advanced atherosclerotic plaques in the GSE43292 dataset. **G** and **H** ROC analysis of the five hub DE-SRGs. ***P < 0.001, ****P < 0.0001, and ns represents no significant difference

Machine learning algorithms have emerged as powerful tools for biomarker discovery and can identify key genes associated with complex diseases such as atherosclerosis. We next employed three machine learning algorithms: LASSO regression (Fig. 2A and B) SVM-RFE (Fig. 2C and D), and RF (Fig. 2E and F) to screen for hub DE-SRGs that may play a role in atherosclerotic plaque progression. Following intersection, six genes (ABI3, CAV1, IRF7, NINJ1, Nox4 and YAP1) were identified as hub DE-SRGs (Fig. 2G). Further validation in GSE43292 confirmed the expression of ABI3, CAV1, NINJ1, Nox4 and YAP1 have remarkable differences (*P* < 0.05) during atherosclerotic plaque progression (Fig. 3A–F) (Table 1). The diagnostic performance of the five hub DE-SRGs was then evaluated by ROC curves, and the area under the ROC curve (AUC) was determined as follows: ABI3 (AUC = 0.778), CAV1 (AUC = 0.804), NINJ1 (AUC = 0.705), Nox4 (AUC = 0.863) and YAP1 (AUC = 0.851) (Fig. 3G). When all five genes were included in one diagnostic model, the AUC value increased to 0.865 (Fig. 3H), suggesting that these hub DE-SRGs have potential as diagnostic markers for atherosclerosis.

**Table 1:** Functional roles of hub DE-SRGs

Genes	Protein	Type	Function
ABI3	ABI gene family member 3	Up	Correlate with cell motility
CAV1	Caveolin-1	Down	May act as a scaffolding protein within caveolar membranes
NINJ1	Ninjurin-1	Up	Homophilic transmembrane adhesion molecule involved in various processes such as inflammation, cell death, axonal growth, cell chemotaxis and angiogenesis
Nox4	NADPH oxidase 4	Down	Constitutive NADPH oxidase generates superoxide intracellularly upon the formation of a complex with CYBA/p22phox. Regulates signaling cascades probably through phosphatases inhibition
YAP1	Transcriptional coactivator YAP1	Down	Transcriptional regulator can act both as a coactivator and a corepressor and is the critical downstream regulatory target in the Hippo signaling pathway that plays a pivotal role in organ size control and tumor suppression by restricting proliferation and promoting apoptosis

### Establishment of a predictive nomogram model for hub DE-SRGs in atherosclerosis progression

**Fig. 4:**
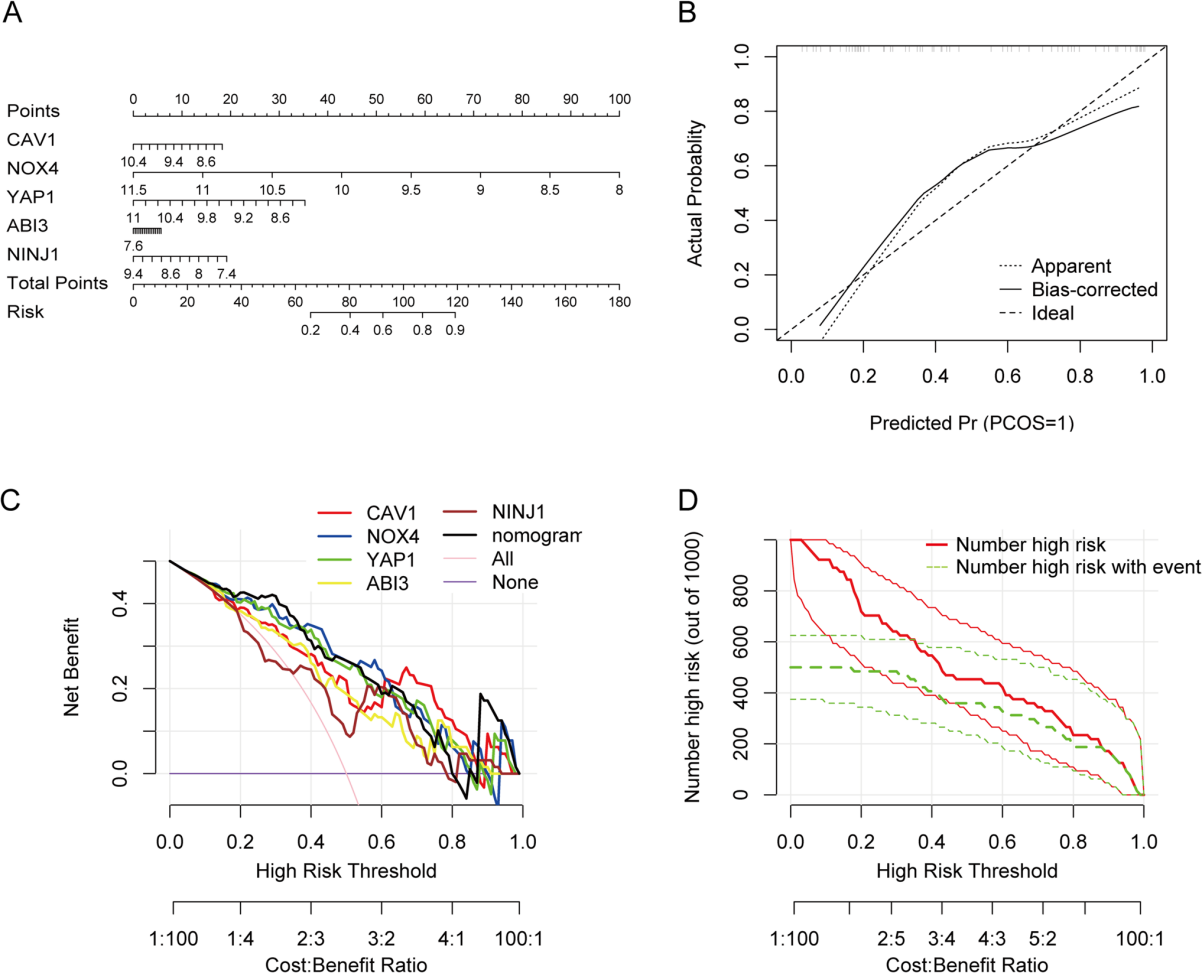
Establishment of a hub DE-SRGs-based nomogram model for atherosclerosis progression diagnosis. **A** Nomogram of multi-variable analysis results to predict the progression of atherosclerosis. Each genetic variable was assigned a corresponding score, and the "total score" was calculated by integrating all predictor variables. **B** The calibration curve showed good agreement between the predicted and actual probabilities of atherosclerosis progression. **C** Decision curve analysis (DCA) shows the clinical benefit of the nomogram model. **D** The clinical impact curves further confirmed the performance of the model, with red curves representing the number of individuals classified as positive (high-risk), and green curves showing the number of true positives at each threshold probability (colour figure online)

To further validate the clinical significance of the identified hub DE-SRGs, a predictive nomogram model was constructed by incorporating the five genes (Fig. 4A). Each gene was assigned a score, and the total score was calculated based on the scores of all characteristic genes. The calibration curve demonstrated that the nomogram ensured a great agreement between prediction and actual observation (Fig. 4B). Furthermore, the decision curve and clinical impact curve analyses confirmed the reliability of the nomogram, highlighting its potential to benefit patients (Fig. 4C and D). Collectively, these findings support the high validity of the selected five genes for predicting atherosclerotic plaque progression.

### Correlation analysis between hub DE-SRGs and immune cells

**Fig. 5:**
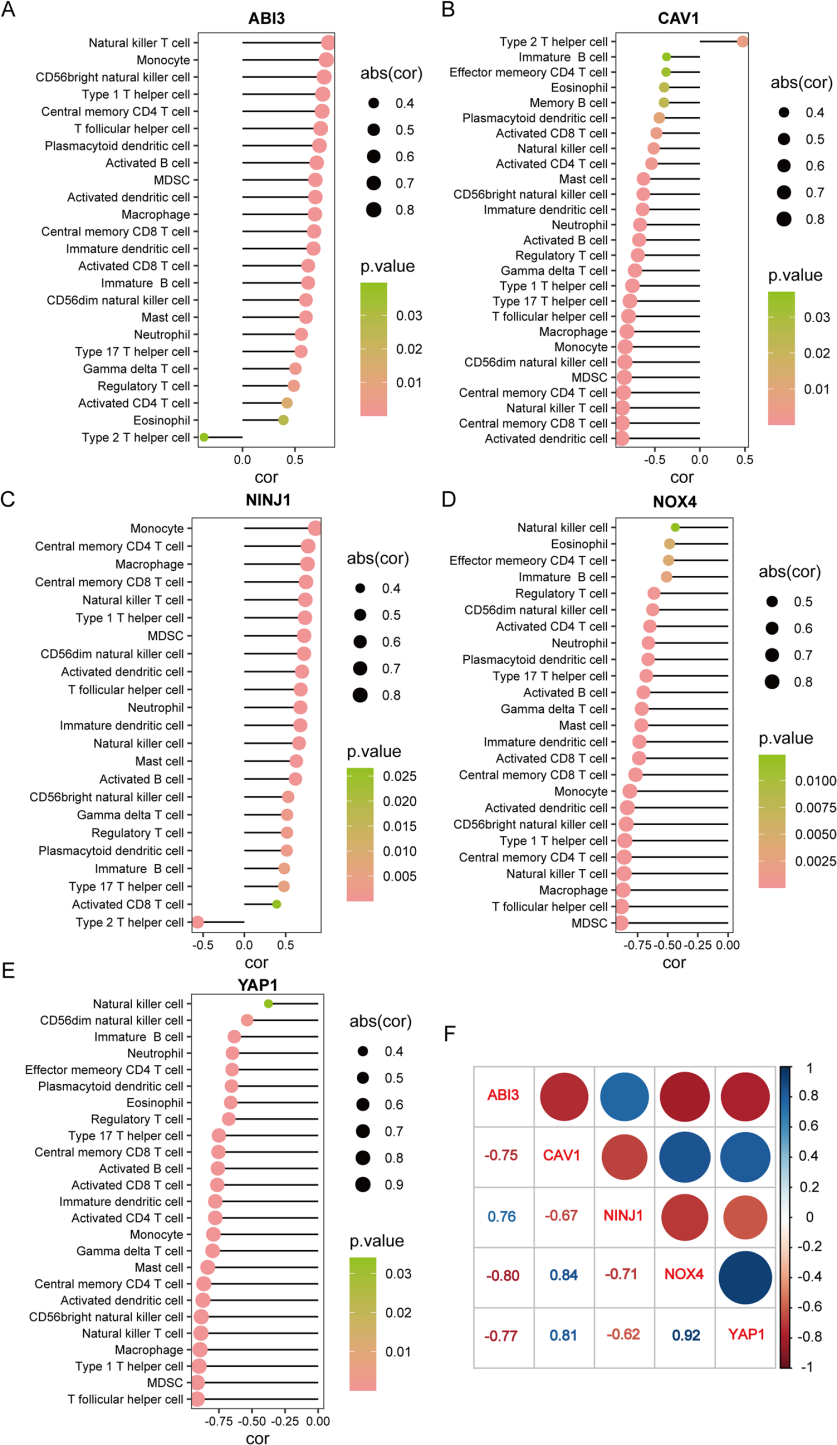
Correlations between the hub DE-SRGs and immune cells. Correlations of **A** ABI3, **B** CAV1, **C** NINJ1, **D** Nox4, and **E** YAP1 with immune cell infiltration. **F** Reciprocal correlations between the five hub DE-SRGs

The accumulation of senescent cells in atherosclerosis is thought to result from deficient immune surveillance and immune stimulatory agents have been shown to increase the removal of these cells [29]. Previous studies have also identified distinct immune cell subpopulations across AS and non-AS [30]. Therefore, we investigated the correlation between the hub DE-SRGs and 28 infiltrating immune cell populations collected by Jia et al.[31] via the ssGSEA method to measure the immune cell enrichment scores. Our correlation analysis revealed that ABI3 had a positive correlation with natural killer T cells, monocytes and CD56bright natural killer cells; CAV1 was found to be negatively correlated with central memory CD4 T cells, natural killer T cells, central memory CD8 T cells and activated dendritic cells; NINJ1 was positively related to monocyte, central memory CD4 T cells, macrophage and central memory CD8 T cells. Additionally, Nox4 and YAP1 were found to have the most significant correlations with macrophages and T follicular helper cells (Fig. 5A–E). Furthermore, our analysis revealed significant reciprocal correlations between the five hub DE-SRGs. Specifically, ABI3/CAV1 (*r* = -0.75), ABI3/NINJ1 (*r*  = 0.76), ABI3/Nox4 (*r*  = −0.8), ABI3/YAP1 (*r* = −0.77), CAV1/Nox4 (*r* = 0.84), CAV1/YAP1 (*r* = 0.81) and Nox4/YAP1 (*r* = 0.92) (Fig. 5F). Taken together, these result suggest that the five hub DE-SRGs play crucial roles in shaping the immune microenvironment which is associated with atherosclerosis progression.

### ABI3 is elevated in advanced atherosclerotic plaques

Consistent with the bioinformatic results, previous studies have illustrated the roles of NINJ1, Nox4, and YAP1 in atherosclerotic plaque development [32–34]. While CAV1, a membrane protein that plays a key role in caveolae formation, has been found to be highly expressed in the endothelium of atherosclerotic plaques, and deficiency of CAV1/caveolae attenuated atherogenesis via suppressing low-density lipoprotein transport across the endothelium and vascular inflammation. Notably, CAV1 is significantly downregulated in vascular smooth muscle cells in atherosclerotic lesions [35]. We next focused on the effect of ABI3 in atherosclerosis progression since it has not been previously investigated. The ABI3 protein displays a complex structure consisting of a Src homology 3 (SH3) domain, a homeobox homology domain, and several proline-rich and serine-rich motifs [36], suggesting that the protein is involved in various biological processes related to gene regulation, signal transduction, and protein–protein interactions.

**Fig. 6:**
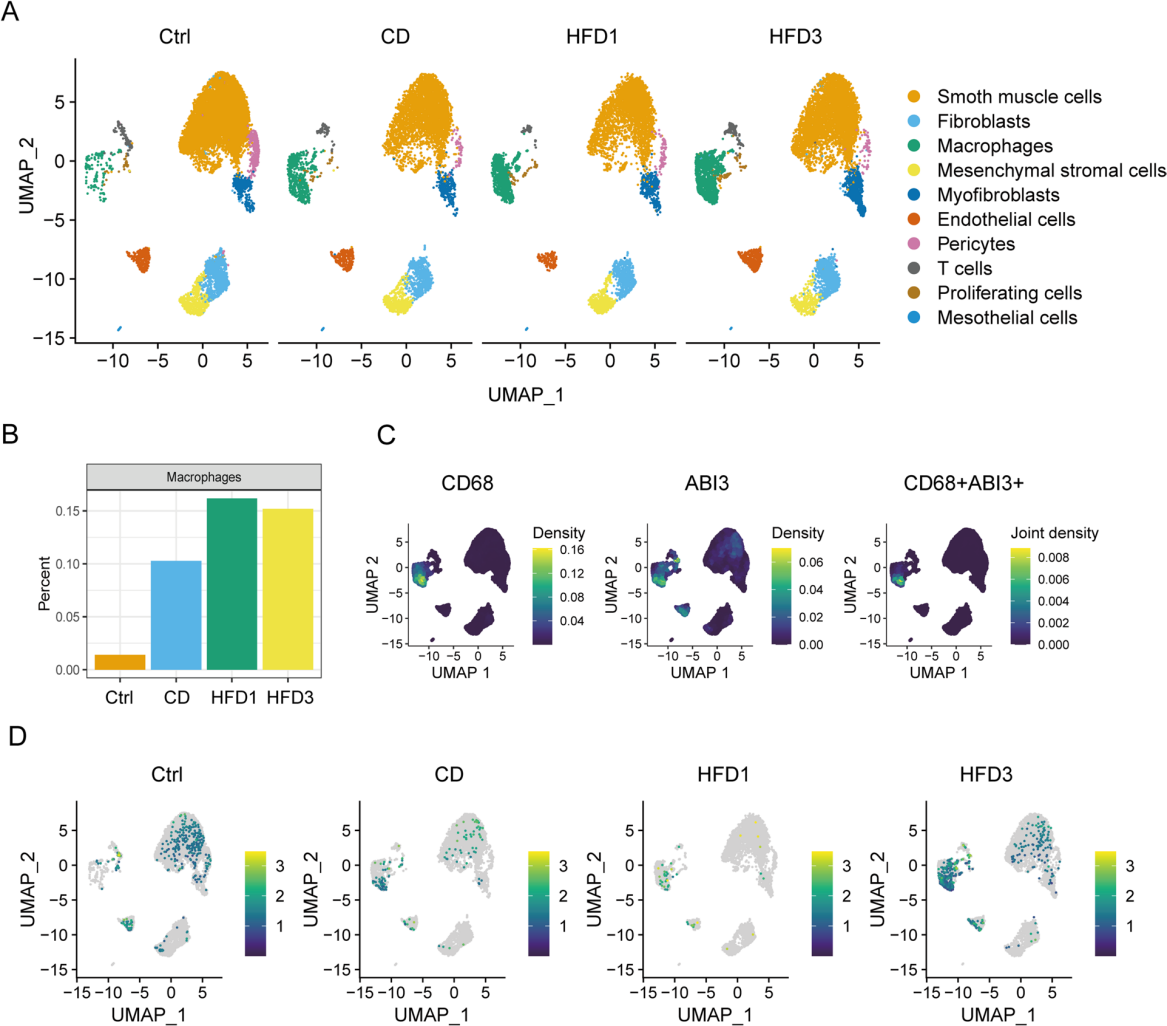
Single-cell analysis re-indicated the high expression of ABI3 in macrophages. **A** UMAP visualization of the ScRNA-seq profiles revealed ten manually annotated clusters; **B** Bar graph shows the proportion of macrophages in the different groups; **C** UMAP visualization displays a co-localization of CD68 and ABI3; **D** UMAP plots show expression distribution of ABI3 mRNA in different cell clusters; Ctrl, C57BL/6J mice fed with chow diet; CD, *Ldlr*^*−/−*^*/Apob*^*100/100*^ mice fed with chow diet; HFD1/HFD3, *Ldlr*^*−/−*^*/Apob*^*100/100*^ mice fed with high fat diet for one/three months to induce early/advanced atherosclerotic lesions for atherosclerotic lesions

To achieve an unbiased and enrichment-free analysis of ABI3 in cell types within healthy and atherosclerotic vascular walls, the ScRNA-seq data GSE205930 containing were acquired from the GEO database [37]. Following quality control and filtering cells, we applied an unbiased clustering approach to categorize cells, resulting in ten distinct subpopulations as visually depicted in the UMAP plot (Fig. 6A). Remarkably, our analysis revealed a significant increase in the proportion of macrophages within the HFD1 (early atherosclerotic lesions) and HFD3 (advanced atherosclerotic lesions) groups compared to other groups, as clearly demonstrated by the bar graph (Fig. 6B). This observation suggests a potential role for macrophages in the progression of atherosclerosis. Furthermore, the UMAP visualization displayed a distinct association between the clusters representing CD68 (a well-established marker for macrophage) and ABI3. This spatial overlap implies that ABI3 is predominantly expressed in macrophages (Fig. 6C), supporting its potential involvement in macrophage-related functions. Consistent with these findings, ABI3 mRNA expression was significantly upregulated in macrophages in atherosclerotic thoracic aorta, moreover, enhanced expression of ABI3 mRNA was observed in the HFD3 group (Fig. 6D), indicating a potential link between ABI3 and the advanced stages of atherosclerosis.

**Fig. 7:**
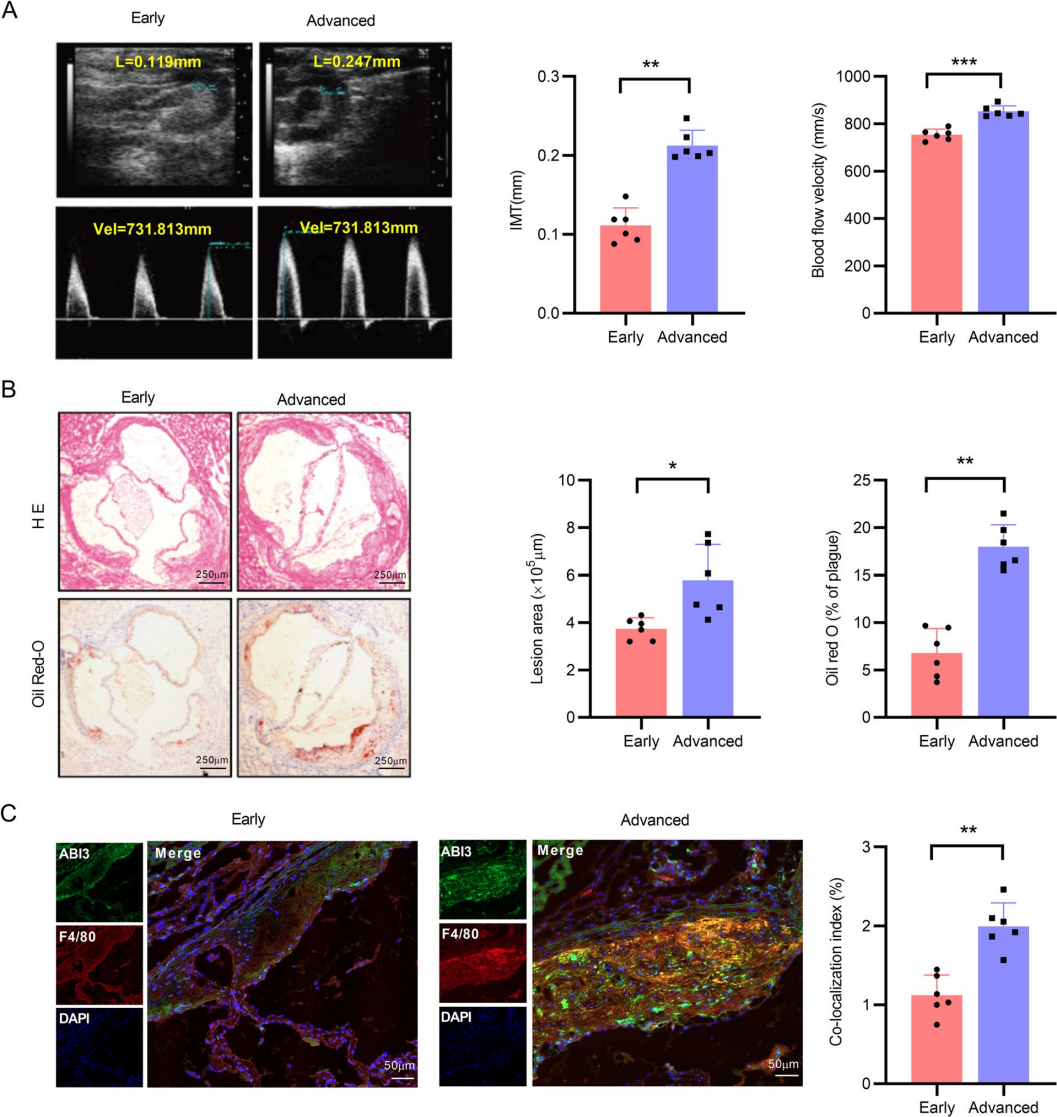
Upregulation of ABI3 in advanced atherosclerosis. *ApoE*^−/−^ mice fed with a high-methionine diet for 4 and 18 weeks to induce the early and advanced atherosclerotic plaques. **A** Representative images and quantification IMT of the aortic root and blood velocity in the ascending aorta of the mice with early or advanced atherosclerotic plaques using UBM. **B** Representative images of cryosectioned aortic roots of the mice with early or advanced atherosclerotic plaques stained by H&E and Oil Red O. Scale bar, 200 μm (*n* = 6). **C** Representative double immunofluorescence staining of ABI3 and F4/80 in aortic roots of the mice with early or advanced atherosclerotic plaques. *P < 0.05, **P < 0.01 (colour figure online)

To further validate these results, we investigated the expression levels of ABI3 in macrophages of advanced atherosclerotic lesions in vivo, *ApoE*^−/−^ mice were fed with a high-methionine diet for 4 or 18 weeks to induce early or advanced atherosclerotic lesions as reported [25]. As shown in Fig. 7A, an elevation of intima-media thickness (IMT) in the aortic root and an increase of blood flow velocity in the ascending aorta were observed in advanced atherosclerotic lesions using ultrasound biomicroscopy (UBM) imaging. Meanwhile, we observed a remarkable increase in plaque formation, and the percentage of lesion area to total aortic area was elevated in advanced lesions, as evidenced by H&E and Oil Red O staining (Fig. 7B). Importantly, a notably increased co-localization of ABI3 with F4/80 (a marker of macrophages) in the aortic root from advanced groups was observed (Fig. 7C), indicating upregulated expression of ABI3 in macrophages from advanced atherosclerotic lesions, which is consistent with our bioinformatic results.

### ABI3 is upregulated in senescent macrophages

**Fig. 8:**
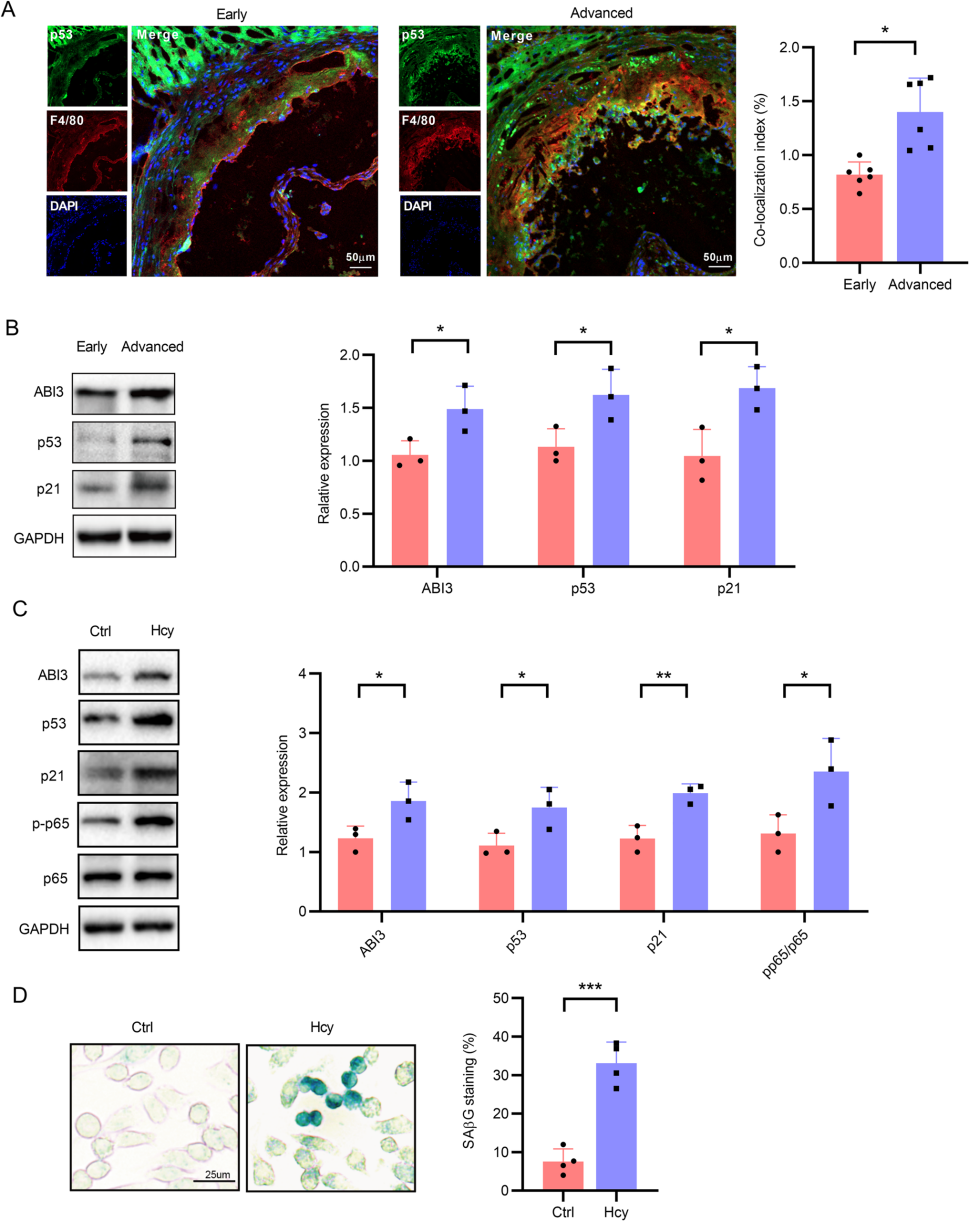
Increased ABI3 expression in senescent macrophages. **A** Representative double immunofluorescence staining of p53 with F4/80 in aortic roots of mice**.** **B** and **C** Measurement protein levels of ABI3 and cellular senescence-related markers p53 and p21 in *ApoE*^−/−^ mice fed with a high-methionine diet and THP-1 monocyte-derived macrophages treated with or without Hcy. **D** Macrophages induced with or without Hcy were stained with SA-β-Gal. Scale bar, 25 μm. *P < 0.05, **P < 0.01, ***P < 0.001

Activation of p53 and subsequent induction of p21 contributes to the development of a senescent phenotype characterized by cell cycle arrest and altered gene expression [38]. Here, we investigated whether ABI3 participates in macrophage senescence induced by Hcy, an independent risk factor for atherosclerosis. We performed immunofluorescence staining for p53 and observed a significant increase in the number of p53 puncta in the macrophages of advanced atherosclerotic plaques, as evidenced by increased co-localization of p53 with F4/80 in the aortic root (Fig. 8A). Consistent with these findings, the protein expression levels of p53, p21, and ABI3 were significantly upregulated in advanced atherosclerotic plaques (Fig. 8B). To establish an Hcy-induced macrophage senescence model, we treated macrophages with Hcy as previously reported [39] and analyzed the expression levels of senescence markers p53 and p21. The results showed a marked upregulation of p53 and p21 expression in macrophages in response to Hcy treatment (Fig. 8C). Meanwhile, senescence-associated-β-galactosidase (SA-β-Gal) staining revealed an increased number of cells positive for β-gal after Hcy treatment (Fig. 8D). Of note, the expression of ABI3 was remarkably enhanced in the Hcy-induced senescent macrophages (Fig. 8C). Collectively, these results indicated that ABI3 might promote macrophage senescence in response to Hcy.

### NF-κB pathway participates in ABI3-induced macrophage senescence

**Fig. 9:**
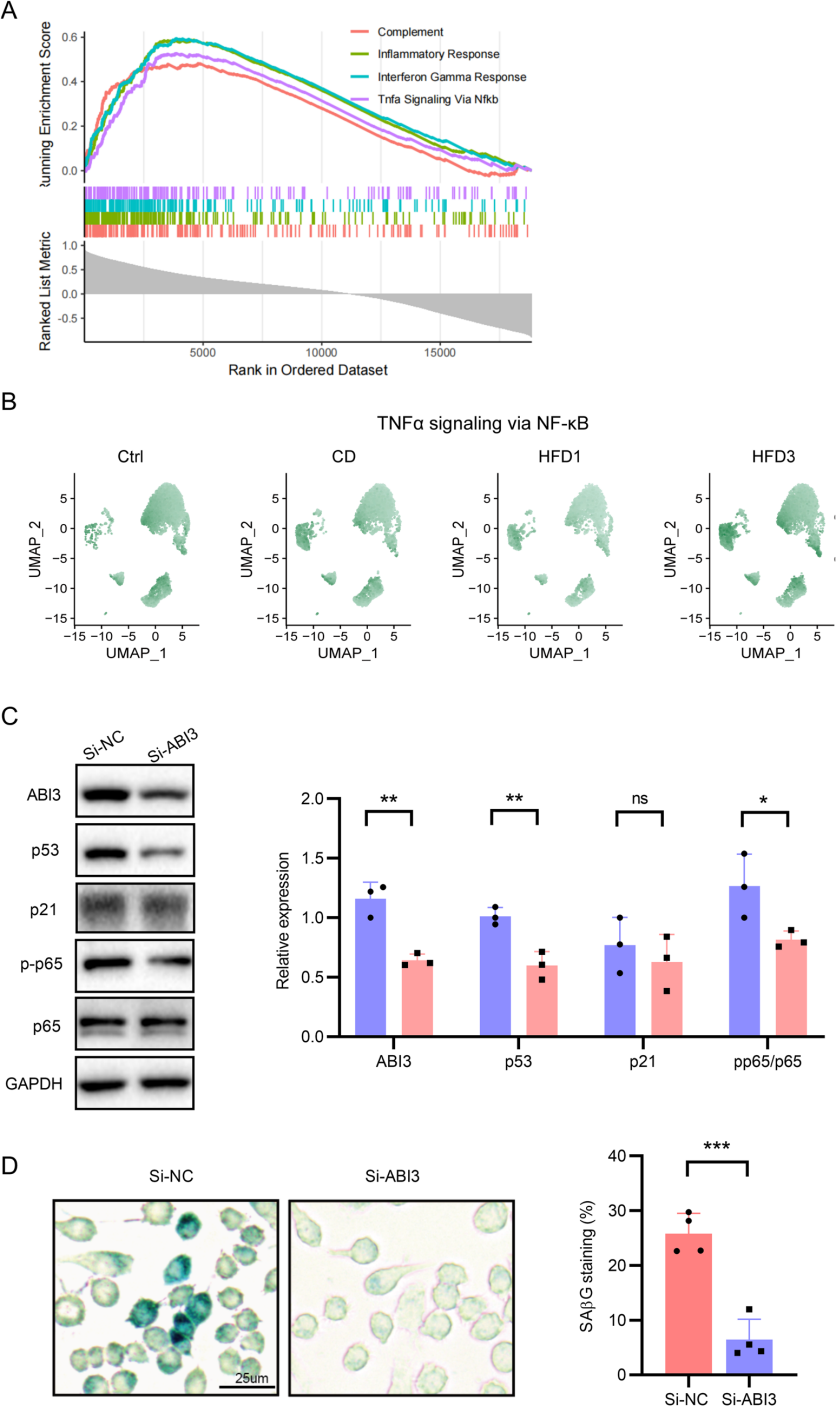
ABI3 facilitates macrophage senescence through NF-κB pathway. **A** GSEA identifies hallmark gene sets that are significantly enriched and activated in the ABI3-high group. **B** UCell score distribution in UMAP space for TNFα signaling via NF-κB pathway was evaluated using the UCell package for the ScRNA-seq data GSE205930. **C** Western blot analysis and quantification of NF-κB p65, p-p65, p53 and p21 in macrophages transfected with Si-NC or Si-ABI3, followed by treatment with Hcy (*n* = 3). **D** Macrophages with indicated treatment were stained with SA-β-Gal. Scale bar, 25 μm. *P < 0.05, **P < 0.01, ***P < 0.001

To further investigate the potential pathways and biological processes associated with ABI3 in macrophage senescence, we performed gene set enrichment analysis (GSEA) based on h.all.v7.4.entrez.gmt collected in Molecular Signatures Database (MSigDB). Our analysis revealed a significant enrichment of the inflammatory response pathway NF-κB in the ABI3-high group compared to the ABI3-low group (Fig. 9A). Subsequently, UCell scoring was applied to further calculate signature scores for individual cells and the scores indicated a high level of activation in the NF-κB pathway within the HFD3 group (Fig. 9B), implying its involvement in the atherosclerosis progression. Furthermore, we knocked down the expression of ABI3 using small interfering RNA (siRNA) in a homocysteine-induced macrophage senescence model, and founded that downregulation of ABI3 expression significantly ameliorated the senescent phenotype of macrophages, as indicated by decreased expression levels of senescence markers p53 and p21, as well as reduced SA-β-Gal staining (Fig. 9C and D). Additionally, knockdown of ABI3 expression resulted in decreased levels of phosphorylated NF-κB p65 (Fig. 9C). Taken together, the results suggest ABI3 might promote macrophage senescence via activation of NF-κB pathway.

## Discussion

As the primary pathological basis of atherosclerotic cardiovascular disease (ASCVD), atherosclerosis, is often life-threatening due to the lack of obvious symptoms at an early stage, and advancing age is a major risk factor for ASCVD. With ageing, immune cells such as monocytes, macrophages and neutrophils are largely affected as the haematopoietic stem cells (HSCs) lose regenerative capacity [40]. Senescent cells accumulation is a major cause of age-related disease and might be driven by oxidative stress, DNA damage, telomere shortening and replicative exhaustion [5]. Senescent foamy macrophages are considered to be deleterious at all stages of atherosclerosis, but the underlying mechanisms remain unclear. In this study, we excavated the SRGs in modulating the atherogenesis progression based on bioinformatics methods, and further functional investigation of hub DE-SRGs provide new insights into selective clearance of senescent cells-based atherosclerosis treatment.

Primarily, 15 DE-SRGs were screened based on two microarray datasets, and five hub DE-SRGs including ABI3, CAV1, NINJ1, Nox4 and YAP1 were identified via integrating three machine learning algorithms. The increased prevalence of NINJ1, a cell surface protein with two transmembrane domains, has been linked to many inflammatory diseases [41]. Previous study have demonstrated that NINJ1 expression was enhanced in atherosclerotic aortas and colocalized with macrophages, and NINJ1 deficiency stimulated inflammatory response of macrophage through activating mitogen-activated protein kinase and blocking PI3K/Akt signaling pathway [32]. Moreover, the expression of NINJ1 promoted p21 levels and G1 cell cycle arrest in Huh-7 hepatoma cells, and NINJ1-overexpressed cells showed increased SAβG activity, suggesting the vital role of NINJ1 in cellular senescence regulation [42]. Studies have found that NADPH oxidases could act as the main source of ROS, and H_2_O_2_-producing NADPH oxidase Nox4 has anti-atherosclerotic functions via regulating compartmental redox states to co-ordinate varied signaling pathways [34, 43, 44]. YAP protein was reported to adapt to atherosclerotic stimuli in a different regulatory manner. Macrophage YAP was up-regulated in human and mouse atherosclerotic plaque compared with non-atherosclerotic vessels, and YAP deficiency in macrophages mitigated atherosclerotic lesion formation [45]. Of note, the levels of phosphorylated YAP (p-YAP) in the aorta of mice fed with a high-fat high-sucrose (HFHS) diet were increased at 2 weeks but decreased at 8 weeks [33]. CAV1, a membrane protein essential for the formation of caveolae, regulates atherogenesis through alleviating transcytosis of low-density lipoprotein and inflammation response independent of increased NO production in endothelial cells. Interestingly, the expression of CAV1 is significantly attenuated in vascular smooth muscle cells in atherosclerotic lesions [35], but highly expressed in the endothelium of atherosclerotic plaques, indicating its complex roles in atherogenesis. ABI3 has been identified as a critical regulator of WASp-family verprolin homologous protein 2 (WAVE2)-induced actin polymerization, which is necessary for cell motility in many functions including immune responses [46]. Studies also have verified that ABI3 contributes to the pathogenesis of age-associated Alzheimer’s disease (AD) [47, 48]. In this study, we established a predictive nomogram based on the five hub DE-SRGs, which displayed a good degree of discrimination and calibration, and could help distinguish the high-risk advanced atherosclerotic plaques in clinical.

Previous research has demonstrated that the ABI3 variant is associated with a range of cardiovascular traits, including hypertension, ischemic heart disease, and coronary atherosclerosis, based on phenome-wide association studies (PheWAS) [49]. In our study, we have further explored the underlying mechanism of ABI3 in atherosclerosis progression. The results illustrated that the expression of ABI3 is significantly upregulated in macrophages of advanced plaques and senescent THP-1-derived macrophages. Furthermore, our findings suggest that the NF-κB signaling pathway plays a critical role in ABI3-mediated macrophage senescence. These findings provide new insights into the mechanism underlying atherosclerosis development and highlight the potential therapeutic utility of targeting the ABI3/NF-κB pathway for the prevention and treatment of this disease.

## Conclusion

In this study, we constructed a diagnostic model consisting of five cellular senescence-associated genes for atherogenesis progression, and identified ABI3 as a macrophage senescence-related protein involved in atherogenesis progression, which propose new preventive and therapeutic strategies of senolytic agents for atherosclerosis.

## Data Availability

The public datasets analyzed in this study can be found in the online GEO database and the accession numbers are listed in the article.

## References

[1] Xu H, Jiang J, Chen W, Li W, Chen Z (2019). Vascular macrophages in atherosclerosis. J Immunol Res.

[2] Ronak Delewi, Hayang Yang, John Kastelein. Textbook of Cardiology, Atherosclerosis. https://www.textbookofcardiology.org/wiki/Atherosclerosis

[3] Lenz T, Nicol P, Castellanos MI, Engel LC, Lahmann AL, Alexiou C, Joner M (2020). Nanoparticle-Enhanced Non-Invasive and Intravascular Molecular Imaging of Atherosclerosis In Vivo. Molecules.

[4] Omran F, Kyrou I, Osman F, Lim VG, Randeva HS, Chatha K (2022). Cardiovascular biomarkers: lessons of the past and prospects for the future. Int J Mol Sci.

[5] Lopez-Otin C, Blasco MA, Partridge L, Serrano M, Kroemer G (2013). The hallmarks of aging. Cell.

[6] Cho JH, Kim EC, Son Y, Lee DW, Park YS, Choi JH, Cho KH, Kwon KS, Kim JR (2020). CD9 induces cellular senescence and aggravates atherosclerotic plaque formation. Cell Death Differ.

[7] Wang H, Fu H, Zhu R, Wu X, Ji X, Li X, Jiang H, Lin Z, Tang X, Sun S (2020). BRD4 contributes to LPS-induced macrophage senescence and promotes progression of atherosclerosis-associated lipid uptake. Aging (Albany NY).

[8] Childs BG, Baker DJ, Wijshake T, Conover CA, Campisi J, van Deursen JM (2016). Senescent intimal foam cells are deleterious at all stages of atherosclerosis. Science.

[9] Cao DJ (2018). Macrophages in cardiovascular homeostasis and disease. Circulation.

[10] Gonzalez-Navarro H, Abu Nabah YN, Vinue A, Andres-Manzano MJ, Collado M, Serrano M, Andres V (2010). p19(ARF) deficiency reduces macrophage and vascular smooth muscle cell apoptosis and aggravates atherosclerosis. J Am Coll Cardiol.

[11] Khanna AK (2009). Enhanced susceptibility of cyclin kinase inhibitor p21 knockout mice to high fat diet induced atherosclerosis. J Biomed Sci.

[12] Jin H, Goossens P, Juhasz P, Eijgelaar W, Manca M, Karel JMH, Smirnov E, Sikkink C, Mees BME, Waring O (2021). Integrative multiomics analysis of human atherosclerosis reveals a serum response factor-driven network associated with intraplaque hemorrhage. Clin Transl Med.

[13] Lee K, Santibanez-Koref M, Polvikoski T, Birchall D, Mendelow AD, Keavney B (2013). Increased expression of fatty acid binding protein 4 and leptin in resident macrophages characterises atherosclerotic plaque rupture. Atherosclerosis.

[14] Ayari H, Bricca G (2013). Identification of two genes potentially associated in iron-heme homeostasis in human carotid plaque using microarray analysis. J Biosci.

[15] Avelar RA, Ortega JG, Tacutu R, Tyler EJ, Bennett D, Binetti P, Budovsky A, Chatsirisupachai K, Johnson E, Murray A (2020). A multidimensional systems biology analysis of cellular senescence in aging and disease. Genome Biol.

[16] Irizarry RA, Hobbs B, Collin F, Beazer-Barclay YD, Antonellis KJ, Scherf U, Speed TP (2003). Exploration, normalization, and summaries of high density oligonucleotide array probe level data. Biostatistics.

[17] Engebretsen S, Bohlin J (2019). Statistical predictions with glmnet. *Clin*. Epigenetics.

[18] Lin X, Li C, Zhang Y, Su B, Fan M, Wei H (2017). Selecting feature subsets based on SVM-RFE and the overlapping ratio with applications in bioinformatics. Molecules.

[19] RColorBrewer S, L.M. Package ‘randomforest’. https://cran.r-project.org/web/packages/randomForest/index.html 2018.

[20] Subramanian A, Tamayo P, Mootha VK, Mukherjee S, Ebert BL, Gillette MA, Paulovich A, Pomeroy SL, Golub TR, Lander ES (2005). Gene set enrichment analysis: a knowledge-based approach for interpreting genome-wide expression profiles. Proc Natl Acad Sci U S A.

[21] Robin X, Turck N, Hainard A, Tiberti N, Lisacek F, Sanchez JC, Muller M (2011). pROC: an open-source package for R and S+ to analyze and compare ROC curves. BMC Bioinformatics.

[22] Harrell FE Jr. R package "rms": Regression modeling strategies. https://cran.r-project.org/web/packages/rms/index.html 2022.

[23] Butler A, Hoffman P, Smibert P, Papalexi E, Satija R (2018). Integrating single-cell transcriptomic data across different conditions, technologies, and species. Nat Biotechnol.

[24] Andreatta M, Carmona SJ (2021). UCell: Robust and scalable single-cell gene signature scoring. Comput Struct Biotechnol J.

[25] Zhou J, Werstuck GH, Lhotak S, de Koning AB, Sood SK, Hossain GS, Moller J, Ritskes-Hoitinga M, Falk E, Dayal S (2004). Association of multiple cellular stress pathways with accelerated atherosclerosis in hyperhomocysteinemic apolipoprotein E-deficient mice. Circulation.

[26] Paigen B, Morrow A, Holmes PA, Mitchell D, Williams RA (1987). Quantitative assessment of atherosclerotic lesions in mice. Atherosclerosis.

[27] Zhao Q, Li S, Li N, Yang X, Ma S, Yang A, Zhang H, Yang S, Mao C, Xu L (2017). miR-34a Targets HDAC1-regulated H3K9 acetylation on lipid accumulation induced by homocysteine in foam cells. J Cell Biochem.

[28] Itahana K, Campisi J, Dimri GP (2007). Methods to detect biomarkers of cellular senescence: the senescence-associated beta-galactosidase assay. Methods Mol Biol.

[29] Ovadya Y, Krizhanovsky V (2014). Senescent cells: SASPected drivers of age-related pathologies. Biogerontology.

[30] Ye Z, Wang XK, Lv YH, Wang X, Cui YC (2022). The integrated analysis identifies three critical genes as novel diagnostic biomarkers involved in immune infiltration in atherosclerosis. Front Immunol.

[31] Jia Q, Wu W, Wang Y, Alexander PB, Sun C, Gong Z, Cheng JN, Sun H, Guan Y, Xia X (2018). Local mutational diversity drives intratumoral immune heterogeneity in non-small cell lung cancer. Nat Commun.

[32] Jeon S, Kim TK, Jeong SJ, Jung IH, Kim N, Lee MN, Sonn SK, Seo S, Jin J, Kweon HY (2020). Anti-inflammatory actions of soluble ninjurin-1 ameliorate atherosclerosis. Circulation.

[33] Liu Y, Li M, Lv X, Bao K, Yu Tian X, He L, Shi L, Zhu Y, Ai D (2022). Yes-associated protein targets the transforming growth factor beta pathway to mediate high-fat/high-sucrose diet-induced arterial stiffness. Circ Res.

[34] Schurmann C, Rezende F, Kruse C, Yasar Y, Lowe O, Fork C, van de Sluis B, Bremer R, Weissmann N, Shah AM (2015). The NADPH oxidase Nox4 has anti-atherosclerotic functions. Eur Heart J.

[35] Ramirez CM, Zhang X, Bandyopadhyay C, Rotllan N, Sugiyama MG, Aryal B, Liu X, He S, Kraehling JR, Ulrich V (2019). Caveolin-1 regulates atherogenesis by attenuating low-density lipoprotein transcytosis and vascular inflammation independently of endothelial nitric oxide synthase activation. Circulation.

[36] Ibanez KR, McFarland KN, Phillips J, Allen M, Lessard CB, Zobel L, De La Cruz EG, Shah S, Vo Q, Wang X (2022). Deletion of Abi3/Gngt2 influences age-progressive amyloid beta and tau pathologies in distinctive ways. Alzheimers Res Ther.

[37] Ord T, Lonnberg T, Nurminen V, Ravindran A, Niskanen H, Kiema M, Ounap K, Maria M, Moreau PR, Mishra PP (2023). Dissecting the polygenic basis of atherosclerosis via disease-associated cell state signatures. Am J Hum Genet.

[38] Yan P, Li Z, Xiong J, Geng Z, Wei W, Zhang Y, Wu G, Zhuang T, Tian X, Liu Z (2021). LARP7 ameliorates cellular senescence and aging by allosterically enhancing SIRT1 deacetylase activity. Cell Rep.

[39] Sun T, Ghosh AK, Eren M, Miyata T, Vaughan DE (2019). PAI-1 contributes to homocysteine-induced cellular senescence. Cell Signal.

[40] Tyrrell DJ, Goldstein DR (2021). Ageing and atherosclerosis: vascular intrinsic and extrinsic factors and potential role of IL-6. Nat Rev Cardiol.

[41] Kayagaki N, Kornfeld OS, Lee BL, Stowe IB, O'Rourke K, Li Q, Sandoval W, Yan D, Kang J, Xu M (2021). NINJ1 mediates plasma membrane rupture during lytic cell death. Nature.

[42] Toyama T, Sasaki Y, Horimoto M, Iyoda K, Yakushijin T, Ohkawa K, Takehara T, Kasahara A, Araki T, Hori M (2004). Ninjurin1 increases p21 expression and induces cellular senescence in human hepatoma cells. J Hepatol.

[43] Langbein H, Brunssen C, Hofmann A, Cimalla P, Brux M, Bornstein SR, Deussen A, Koch E, Morawietz H (2016). NADPH oxidase 4 protects against development of endothelial dysfunction and atherosclerosis in LDL receptor deficient mice. Eur Heart J.

[44] Miller FJ (2015). Nox4 NADPH oxidase: emerging from the veil of darkness. Eur Heart J.

[45] Liu M, Yan M, Lv H, Wang B, Lv X, Zhang H, Xiang S, Du J, Liu T, Tian Y (2020). Macrophage K63-linked ubiquitination of YAP promotes its nuclear localization and exacerbates atherosclerosis. Cell Rep.

[46] Sekino S, Kashiwagi Y, Kanazawa H, Takada K, Baba T, Sato S, Inoue H, Kojima M, Tani K (2015). The NESH/Abi-3-based WAVE2 complex is functionally distinct from the Abi-1-based WAVE2 complex. Cell Commun Signal.

[47] Conway OJ, Carrasquillo MM, Wang X, Bredenberg JM, Reddy JS, Strickland SL, Younkin CS, Burgess JD, Allen M, Lincoln SJ (2018). ABI3 and PLCG2 missense variants as risk factors for neurodegenerative diseases in Caucasians and African Americans. Mol Neurodegener.

[48] Karahan H, Smith DC, Kim B, Dabin LC, Al-Amin MM, Wijeratne HRS, Pennington T, Viana di Prisco G, McCord B, Lin PB (2021). Deletion of Abi3 gene locus exacerbates neuropathological features of Alzheimer's disease in a mouse model of Abeta amyloidosis. Sci Adv.

[49] Li QS, Tian C, Hinds D, Me Research T, Seabrook GR (2020). The association of clinical phenotypes to known AD/FTD genetic risk loci and their inter-relationship. PLoS ONE.

